# Health Utilities in Alzheimer's Disease: A Survey of US Patients and Caregivers

**DOI:** 10.1002/alz70860_100502

**Published:** 2025-12-23

**Authors:** Pei‐Jung Lin, Abigail G Riley, Patricia G Synnott, Theresa Frangiosa, Amber Roniger, Peter J Neumann, Joshua T Cohen

**Affiliations:** ^1^ Tufts Medical Center, Boston, MA, USA; ^2^ Tufts University School of Medicine, Boston, MA, USA; ^3^ UsAgainstAlzheimer's, Washington, DC, USA

## Abstract

**Background:**

The introduction of new Alzheimer's disease (AD) treatments in the US necessitates updated patient and caregiver health utility estimates for economic evaluations.

**Method:**

We conducted a web‐based survey to characterize health‐related quality of life (HRQoL) of US adults with mild cognitive impairment (MCI) or AD, and adults who self‐identified as a current caregiver of someone with MCI or AD. We measured health utilities using the EuroQol EQ‐5D‐5L and Quality of Life in AD (QoL‐AD) instruments. The survey sample was drawn from UsAgainstAlzheimer's A‐LIST® online cohort. Individuals with MCI or mild Alzheimer's dementia self‐reported their utilities. Caregivers randomly received either a proxy survey to complete on behalf of the person with moderate to severe AD they cared for, or a caregiver survey that asked them to self‐report their own utilities. We computed mean utility scores for patients and caregivers separately, stratified by disease severity (MCI, mild, moderate, and severe AD) and care setting (community and nursing home).

**Result:**

The final analytic sample comprised 241 patient responses (patient self‐reports: *n* = 105; caregiver proxy‐reports: *n* = 136) and 176 caregiver responses (i.e., caregiver self‐reports). Patient EQ‐5D‐5L scores decreased monotonically as disease severity increased. Health utilities among community‐dwelling individuals with MCI or AD were as follows: 0.77 (MCI), 0.68 (mild), 0.49 (moderate), and 0.22 (severe). EQ‐5D‐5L values were generally lower for individuals residing in nursing homes (0.04 to 0.78) compared to those in community settings (0.22 to 0.77). Patients’ QoL‐AD scores did not exhibit a consistent association with disease severity. Caregivers’ EQ‐5D‐5L scores also did not exhibit a monotonic trend with the patient's disease severity, although caregiver utilities were generally higher for those caring for someone in a nursing home than for those caring for patients in the community.

**Conclusion:**

Our findings showed that health utilities decreased with AD severity, with lower utilities observed in nursing home residents versus community patients. Caregivers of nursing home patients with AD reported higher utilities than those caring for community‐dwelling patients. Our results contribute to improving AD economic evaluations by reflecting more contemporary populations and facilitating value assessments of novel therapies that delay the progression from MCI to more severe disease stages.

